# Study on NO_2_ Barrier Properties of RTV Silicone Rubber by Incorporation of Functional Graphene Oxide

**DOI:** 10.3390/ma16051982

**Published:** 2023-02-28

**Authors:** Zhen Huang, Jinshuai Zhang, Zheng Wang, Xiangyang Peng, Jiapeng Fang, Chunqing He, Pengfei Fang

**Affiliations:** 1 Guangdong Key Laboratory of Electric Power Equipment Reliability, Electric Power Research Institute of Guangdong Power Grid Co., Ltd., Guangzhou 510080, China; 2 Key Laboratory of Nuclear Solid State Physics Hubei Province, School of Physics and Technology, Wuhan University, Wuhan 430072, China

**Keywords:** resistibility, functional graphene oxide, silicone rubber, electrochemical performance

## Abstract

In this study, functional graphene oxide (f-GO) nanosheets were prepared to enhance the NO_2_ resistibility of room-temperature-vulcanized (RTV) silicone rubber. A nitrogen dioxide (NO_2_) accelerated aging experiment was designed to simulate the aging process of nitrogen oxide produced by corona discharge on a silicone rubber composite coating, and then electrochemical impedance spectroscopy (EIS) was used to test the process of conductive medium penetration into silicone rubber. After exposure to the same concentration (115 mg·L^−1^) of NO_2_ for 24 h, at an optimal filler content of 0.3 wt.%, the impedance modulus of the composite silicone rubber sample was 1.8 × 10^7^ Ω·cm^2^, which is an order of magnitude higher than that of pure RTV. In addition, with an increase in filler content, the porosity of the coating decreases. When the content of the nanosheet increases to 0.3 wt.%; the porosity reaches a minimum value 0.97 × 10^−4^%, which is 1/4 of the porosity of the pure RTV coating, indicating that this composite silicone rubber sample has the best resistance to NO_2_ aging.

## 1. Introduction

Silicone rubber is widely used in aerospace [1], energy storage [2], power grid [3], medical and flexible electronic equipment [4,5] due to its excellent properties, such as high flexibility, good thermal stability, excellent corrosion resistance, electrical insulating, high abrasion resistance [6], mechanical strength and hydrophobicity. Silicone rubber mainly includes high-temperature-vulcanized silicone rubber (HTV) and room-temperature sulfur silicone rubber (RTV), both of which have a large range of applications in the electric power industry [7] because of their hydrophobicity and hydrophobicity recovery, as well as excellent anti-flashover performance [8]. However, silicone rubber may lose hydrophobicity in practical working environments, such as discharge [9], ultraviolet light, high temperature and high humidity [10,11]. In particular, many oxidizing gases such as NO_2_ are produced in the discharge process. The destruction of silicone rubber by NO_2_ will lead to molecular oxidative decomposition, which affects hydrophobicity and water resistance [12,13,14]. If the silicone rubber is permeated by water, the outer insulating protective layer will discharge and flashover, which will lead to equipment damage and power outages. Therefore, it is a tremendous challenge to effectively prevent NO_2_ from damaging silicone rubber and improve the antioxidant property of silicone rubber [15,16,17].

The effect of conventional fillers (aluminum trihydroxide ATH and silica) on the properties of silicone rubber has been studied. Mahmoood et al. [18] studied the aging behavior of RTV-SiR loaded with nano-silica and micron ATH, manufactured material samples of different specifications and subjected to various ambient pressures at AC and bipolar DC voltages for 9000 h in two specially designed weather chambers. The silicone rubber doped with nano-sized silica filler particles showed better aging resistance compared with the micro-ATH-filled silicone rubber. Relevantly, two-dimensional (2D) nanosheet materials, such as C_3_N_4_ [19], clay [20], carbon nanofillers [21], BN [22] and GO [23], have been widely used to improve the various properties of polymers [24], such as the gas barrier properties of polymer materials, due to their high-aspect-ratio characteristics. Most of all, GO is a 2D lamellar material composed of sp^2^ hybrid carbon atoms with a large theoretical surface area (~2630 m^2^·g ^−1^), which provides excellent physical barrier properties. The addition of GO in polymer composites also makes it extremely low in permeability to O_2_, H_2_O or CO_2_. For example, Layek et al. [23] fabricated layer-structure graphene oxide/polyvinyl alcohol nanocomposite-coated polyethylene terephthalate (PET) films by interacting via H bonding. The obtained GO composite films show a dramatic enhancement in hydrogen gas barrier properties, which shows a 95% decrease in the permeability coefficient compared to uncoated PET. Tzeng et al. [25] created chitosan-poly(acrylic acid)/chitosan/ graphene oxide quadlayers (CH/PAA/CH/GO QLs) with a highly ordered nanobrick wall structure via layer-by-layer deposition. A five CH/PAA/CH/GO QL assembly exhibits very low oxygen permeability (3.9 × 10^−20^ cm^3^·cm·cm^−2^·Pa^−1^·s^−1^) that matches SiO_x_ barrier coatings. Yu et al. [26] prepared TiO_2_-GO nanocomposites using 3-minopropyltriethoxysilane as a linking reagent, which possess a sheet structure with greater interlayer spacing. The hybrids not only have excellent exfoliation and dispersion in epoxy resin, but also obviously enhanced H_2_O barrier properties and anti-corrosion performance of epoxy coatings at a low content (2 wt.%). Zhang et al. [27] observed an extremely low water contact angle (mean ≈ 30°), which clearly confirmed the inherent hydrophilicity of primitive graphene, after excluding the interference of substrate and pollutants. This hydrophilicity was found to be caused by charge transfer between graphene and water based on H-π interactions. Graphene oxide (GO), as the most important graphene derivative, has abundant oxygen functional groups, huge specific area and strong hydrophilicity, which makes GO easy to aggregate and difficult to separate. Ramezanzadeh et al. [28] found that polyisocyanate-grafting GO enhanced its compatibility and dispersion in the polyurethane resin, and it significantly improved the water barrier performance and corrosion resistance. Wan et al. [29] also found that the surface functionalization of diglycidyl ether of bisphenol-A layer can effectively improve the compatibility and dispersion of GO sheets in an epoxy matrix. However, to date, there are few reports on GO applied to improve the NO_2_ gas barrier and antioxidant properties of polymer composites.

In this work, surface-modified GO nanosheets (f-GO) were prepared using tetraethoxysilane (TEOS) and aminopropyltriethoxysilane (APTES) via a convenient sol–gel method. The effect of f-GO on the NO_2_ resistibility for RTV coatings was evaluated by water diffusion behavior via electrochemical impedance spectroscopy (EIS) of f-GO/RTV nanocomposites, which was treated by oxidation damage from NO_2_ gas.

## 2. Materials and Methods

### 2.1. Materials

Hydroxyl-terminated polydimethylsiloxane (OH-PDMS, 50–100 thousand relative molecular weight) was obtained form Jinan Guobang Chemical Co., Ltd., Jinan, China. The OH-PDMS is colorless transparent liquid, and its viscosity is between 7000 and 120,000 (25 °C), volatile content ≤2%(150 °C, 3 h) and surface vulcanization time is less than or equal to 2 h. TEOS and APTES were obtained from Shanghai Macklin Biochemical Technology Co., Ltd., Shanghai, China. The chemical formula of TEOS is C_8_H_20_O_4_Si, and its purity is as follows: silica: 38.0~42.0%. The chemical formula of APTES is C_9_H_23_NO_3_, and its purity is 98%. SiSilica (SiO_2_) was obtained from Guangzhou GBS High-Tech & Industry Co., Ltd., Guangzhou, China. The scale of SiO_2_ is nano level. Aluminum hydroxide (ATH) was obtained from Aluminum Corporation of China. The scale of ATH is micron level, which is 90-mesh grain size.

### 2.2. Preparations

**Figure 1:**
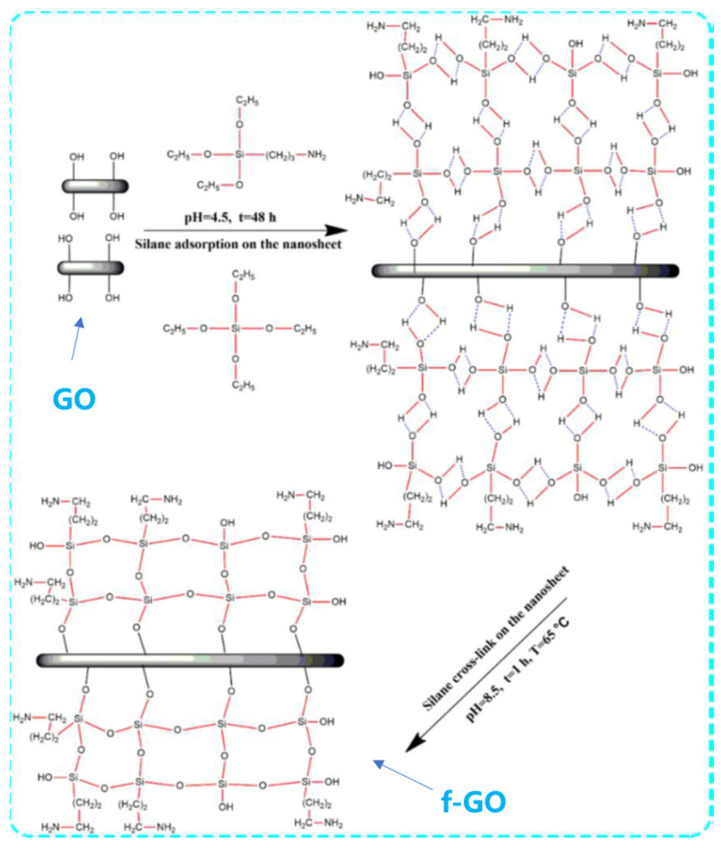
A synthesis diagram of functionalized GO nanosheets.

Functionalized graphene oxide (f-GO) preparation: GO was prepared by using a modified Hummers’ method [18]. As shown in Figure 1, a certain amount of GO was dispersed into a mixture of TEOS and APTES (7:3, wt.%), mechanically stirred for 1 h and then sonicated for 2 h. We then adjusted the pH value to 4.5 with glacial acetic acid and deposited for 48 h. Then, the pH was adjusted to 8.5 using 2.5 wt.% NaOH solution, and the gel was placed in a water bath at 65 °C. The final product was dried in a freeze-drying oven to obtain f-GO.

Preparation of f-GO/RTV nanocomposite coatings: The OH-PDMS, f-GO and other inorganic fillers (ATH, SiO_2_ et al.) were physically mixed at 110 °C for 5 h. After the temperature was cooled to 40 °C, the other additives were added and the system mixed continuously for 1 h. Then, the f-GO/RTV nanocomposite was spanned to ITO glasses and cured at room temperature for 24 h. The coating thickness was 45 ± 2 μm.

Oxidation damage of oxidizing gas NO_2_: The prepared coating was subjected to NO_2_ aging damage test at room temperature with a self-made device. The volume of the instrument was 2.5 L. In order to ensure that the experiment was carried out in a dry environment, anhydrous CaCl_2_ was added to the bottom of the instrument to dry for 48 h, and then the sample was put into the aging device. In the experiment, quantitative copper was used to react with nitric acid to control the concentration of NO_2_.

### 2.3. Characterization

The morphologies of f-GO were characterized by scanning electron microscopy (SEM, Hitachi S-4800, FEI Company, Hillsboro, OR, USA). The chemical bonding states of the f-GO nanosheets were analyzed by Fourier-transformed infrared spectroscopy (FTIR, Nicolet iS10, Thermo Electron Corporation, Waltham, MA, USA) and X-ray photoelectron spectroscopy (XPS, ESCALAB 250Xi, Thermo Fisher Scientific, Shanghai, China).

The EIS test instrument is CS310 Electrochemical workstation (Coster, Wuhan, China); the test software is Electrochemical & Corrosion Studio v5.0. During the EIS test, nanocomposite coatings were immersed in 3.5 wt% NaCl solution and the actual working area of the sample is 1 cm^2^. The testing amplitude sinusoidal voltage was 20 mV and the current range was 20 nA. The testing frequency ranged from 10^5^ Hz to 10^−1^ Hz and the number of test points was 10 within each order of magnitude. The bandwidth capacitance setting is as follows: when frequency is larger than 10 Hz, bandwidth capacitance is 22 pF; in other cases, the bandwidth capacitance is 470 pF.

## 3. Results and Discussion

**Figure 2:**
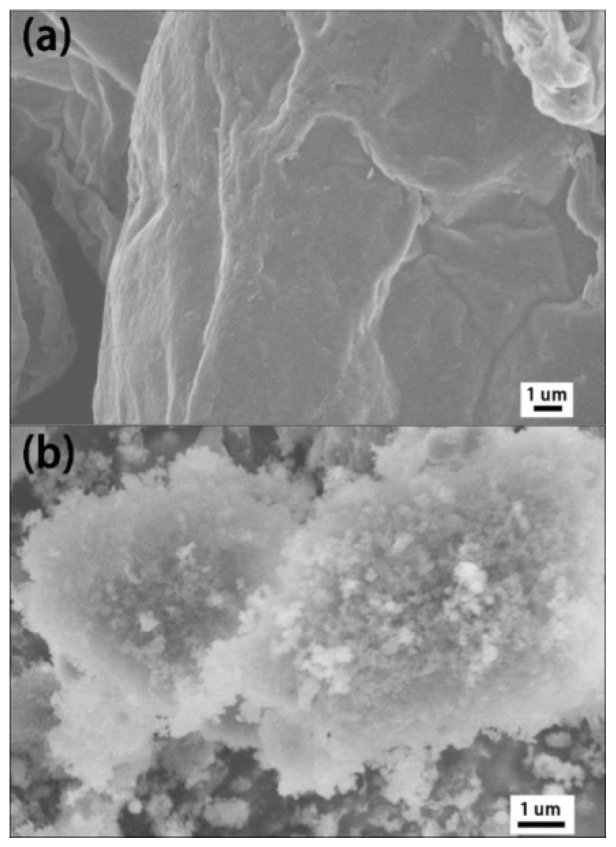
SEM images of GO nanosheets: (**a**) before surface modification GO, (**b**) after surface modification f-GO (**b**).

Figure 2a shows the unmodified GO nanosheet. Due to the interaction of van der Waals forces between the lamellae, the unmodified GO exhibits a large aggregate state with a smooth surface. After silane surface modification, the surface of the f-GO nanosheet is no longer smooth, but fluffy granular objects appear on its surface. It is speculated that silicone nanoparticles are formed by condensation of hydroxyl on the surface of silane and GO.

**Figure 3:**
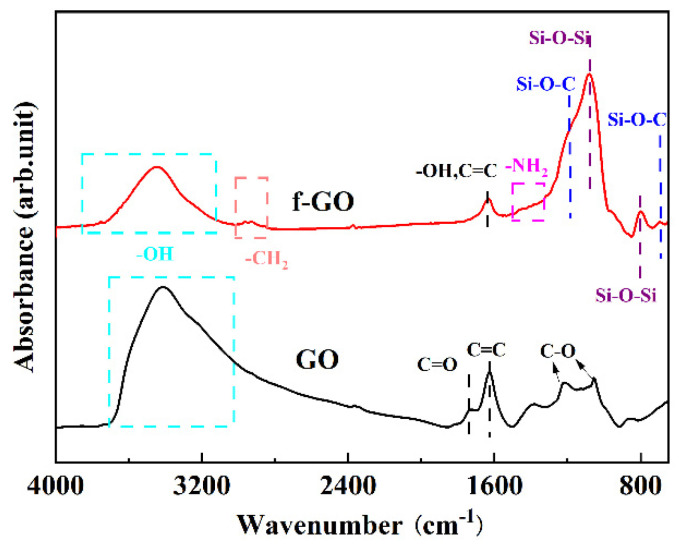
ATR-FTIR spectra of GO and f-GO nanosheets.

ATR-FTIR was used to analyze the chemical composition of GO surface before and after modification to evaluate its modification and structure maintenance. In Figure 3, the characteristic peaks before modification mainly include −OH at 3400 cm^−1^, C=O at 1720 cm^−1^, C=C at 1690 cm^−1^ and C−O at 1230 cm^−1^ [30,31]. The results show that there are abundant oxygen-containing groups on the surface of GO, providing active sites for sol–gel surface modification. After the surface modification of silane sol–gel, C=C was still observed in the spectrogram, indicating that the silane modification did not destroy the structure of GO. In addition, some new characteristic peaks appear in the spectrogram. Among them, −CH_2_, located at 2960 cm^−1^, is derived from methylene group in APTES. The peak around 1500 cm^−1^ belongs to −NH_2_ of APTES [26,32]. In addition, C−O−Si characteristic peaks of asymmetric bending vibration at 1124 cm^−1^ and 694 cm^−1^ and Si−O−Si peak [26] of asymmetric vibration at 1090 cm^−1^ also appear. The presence of C−O−Si bonds at 694 and 1124 cm^−1^ confirms that the hydrolysate reacts with and binds covalently to the −OH group of GO nanosheets via the silane-hydrolyzed ethoxy group.

**Figure 4:**
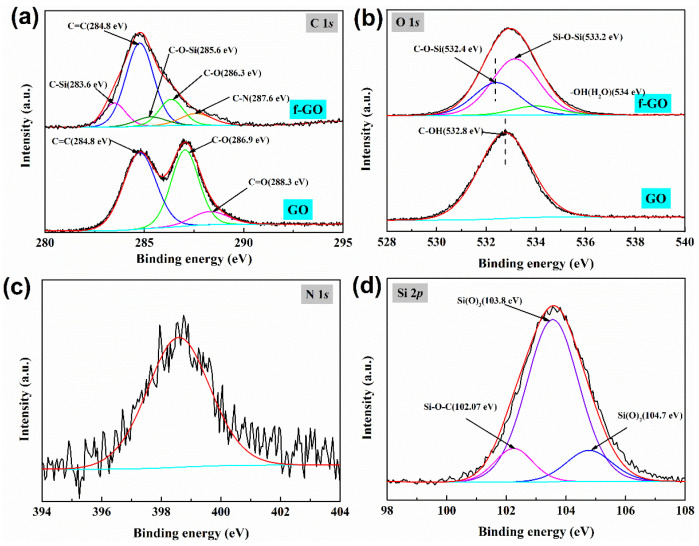
XPS analysis of GO and f-GO nanosheets: C 1s (**a**), O 1s (**b**), N 1s (**c**), Si 2p (**d**).

As shown in Figure 4, XPS was used to analyze the surface chemical composition of GO before and after modification. As shown in Table 1, the element composition of GO is C and O (66.56 at%: 33.44 at%). For f-GO nanosheets, the elemental compositions are C, N, O and Si (32.38 at%: 1.9 at%: 39.57 at%: 26.15 at%). The presence of silicon and nitrogen indicates that silane was successfully presented on the sheet surface. The appropriate combination of Lorentz function and Gaussian function is used to distinguish the C 1s, N 1s, O 1s and Si 2p bonding states of f-GO nanosheets and GO. High-resolution energy spectrum analysis showed that C 1s of GO was mainly composed of C=C, C−O and C=O bonds located at 284.8, 286.9 and 288.3 eV before modification. After sol–gel modification, in addition to the above three peaks, three new peaks appeared at 283.6, 285.6 and 287.6 eV, which were, respectively, attributed to C−Si, C−O−Si and C−NH_2_ [33]. The continuous presence of C=C, C−O and C=O peaks indicates that silane modification has no effect on the structure of GO. The appearance of C−Si and C−O−Si indicates that the hydroxyl group on the surface of GO is condensed with the hydroxyl group of silanes to form a covalent bond. However, for the peak splitting of O 1s, it was found that the O of GO was mainly hydroxyl oxygen at 532.8 eV [30]. After silane modification, a new peak belonging to C−O−Si appeared at 532.4 eV in the direction of low binding energy, which may be due to the transformation of C−O−H structure on graphene surface into C−O−Si structure during the condensation process of hydroxyl groups on silane and graphene surface. Since H is more electronegative than silicon, the bonding position of oxygen moves 0.4 eV toward the direction of low binding energy [34]. At the same time, Si−O−Si appeared in the oxygen peak, which also indicated that silicone appeared on the surface of graphene, which was consistent with the results of FTIR and SEM. At the same time, N element was also detected on the surface of the sample, which was mainly derived from aminopropyl trethoxy silane. However, for silicon, it mainly consists of three peaks, namely Si-O-C, Si(O)_2_ and Si(O)_3_, located at 120.07, 103.8 and 104.7 Ev [35], respectively, which again indicates that successful grafting condensation of silane and graphene occurs, rather than simple mechanical mixing.

**Figure 5:**
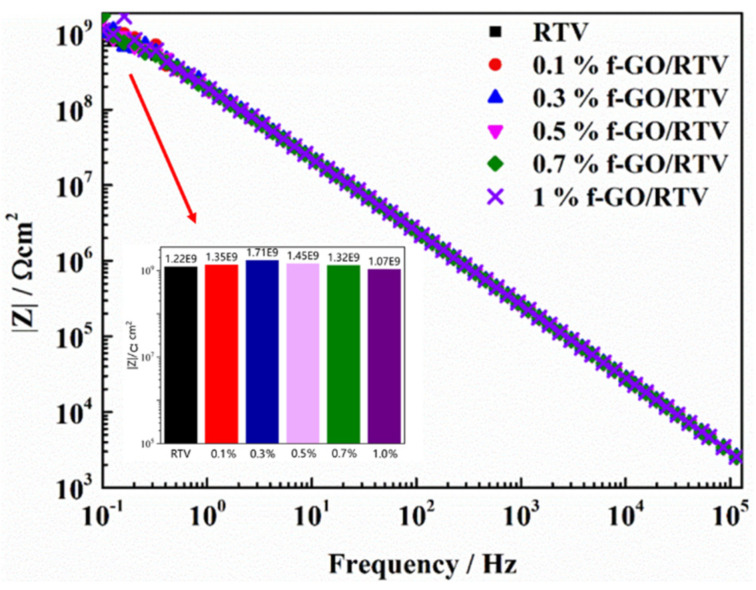
Impedance modulus value of f-GO/RTV silicone rubber nanocomposite coatings with different filler contents after immersing in 3.5 wt.% NaCl solution for 96 h.

To quantitatively analyze the NO_2_ resistibility of the f-GO/RTV nanocomposite, the coatings with different f-GO contents were evaluated by EIS after oxidation by NO_2_. Before NO_2_ aging, the impedance spectra of RTV nanocomposite coatings filled with different contents of f-GO nanosheets are shown in Figure 5. After immersing in NaCl solution for 96 h, the impedance modulus |Z| of each sample in the Bode diagram remained at 10^9^ Ω cm^2^, and the phase angle was basically maintained at 90° in the whole frequency range, which means that the sample fully exhibited capacitive property and the aqueous solution failed to penetrate into the sample, indicating that the addition of fillers had no effect on the microstructure and water resistance of the sample. Even after 96 h immersion, the coating remained intact and the sample can still be regarded as a pure capacitor. To quantitatively analyze the tolerance of functionalized f-GO/RTV nanocomposite coatings to oxidizing gas NO_2_, EIS was used to evaluate the water barrier properties of pure RTV and f-GO/RTV nanocomposite coatings after NO_2_ aging.

**Figure 6:**
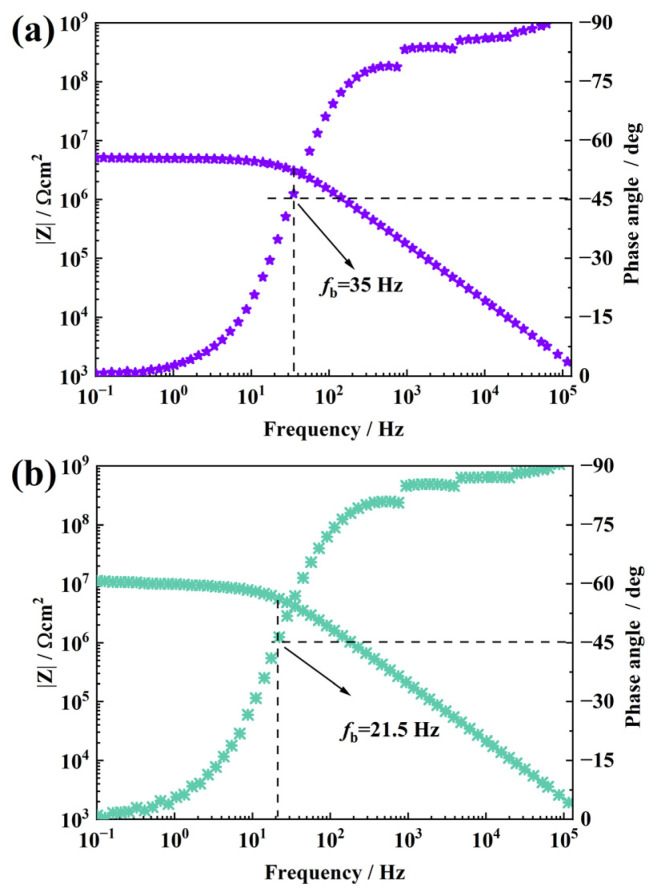
Bode plots of RTV coatings (**a**) and 0.1 wt.% f-GO/RTV nanocomposite coatings (**b**) after NO_2_ oxidation.

As shown in Figure 6a, the water barrier performance of pure RTV decreased significantly after NO_2_ aging. After soaking for 96 h, the impedance modulus decreased significantly compared with that of the unaged sample, which decreased by three orders of magnitude to about 5.14 × 10^6^ Ω·cm^2^. It shows that after NO_2_ aging, many water diffusion channels are formed in the sample, and the diffusion of electrolyte solution in the sample causes a decrease in impedance modulus. At the same time, it is observed in the frequency and angle diagram that the frequency of the sample at 45° increases significantly. The frequency at 45° is called the breakpoint frequency $f_{b}$, and its value can qualitatively analyze the stratification of the coating [36,37]. The larger the breakpoint frequency, the more serious the stratification. For pure RTV, $f_{b}$ was about 35 Hz after NO_2_ aging, indicating that aging caused a certain degree of stratification. After filling with 0.1 wt.% f-GO nanosheets, the impedance modulus of the sample increased to about 1.13 × 10^7^ Ω·cm^2^ compared with pure RTV, indicating that the filling of nanosheets slowed down the diffusion of NO_2_ gas in the coating to a certain extent. At the same time, $f_{b}$ decreases to 21.5 Hz. The results show that after aging for the same time, the stratification of the sample decreases and the integrity of the sample is maintained. However, the breakpoint frequency of the sample is still high, and there is still serious stratification, but the damage is reduced, so it is necessary to further increase the filling amount of the nanosheet.

**Figure 7:**
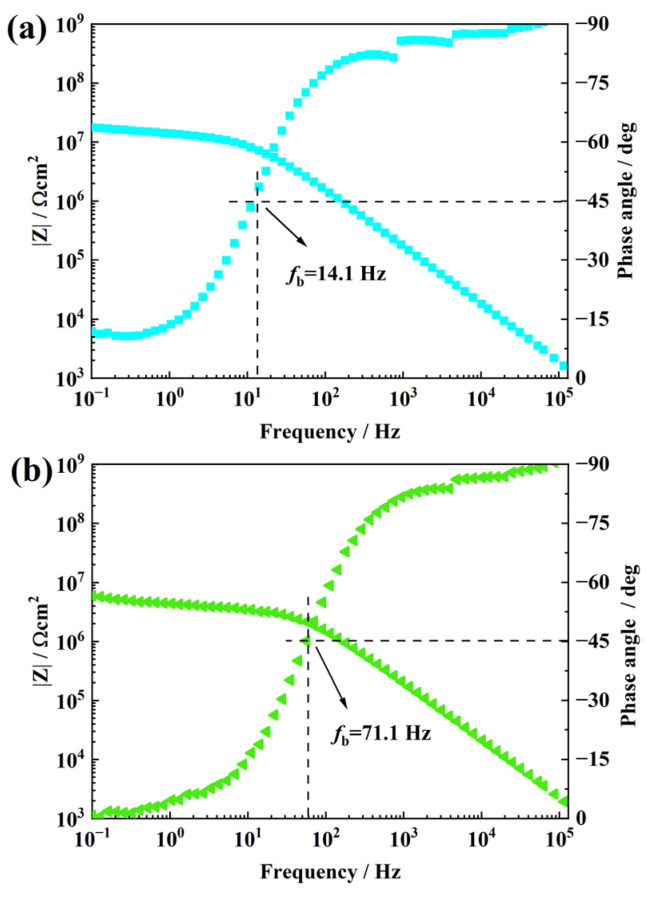
Bode plots of RTV nanocomposite coatings, respectively, filled with 0.3 wt.% (**a**) and 0.5 wt.% (**b**) f-GO nanosheets after NO_2_ oxidation.

Figure 7a shows the impedance spectrum of the sample filled with 0.3 wt.% f-GO nanosheets after NO_2_ aging for 24 h and soaking for 96 h. The impedance modulus value is about 1.8 × 10^7^ Ω·cm^2^, indicating that the impedance modulus value continues to increase with the increase in filler content. The impedance modulus of this sample is an order of magnitude higher than that of pure RTV, and capacitive behavior is observed over a wide frequency range (10^2^ to 10^5^ Hz). At the same time, $f_{b}$ also decreased significantly, which was about 14.1 Hz, indicating that the increase in the content of functionalized nanosheets greatly delayed the diffusion of NO_2_ gas inside the coating. The extension of the gas diffusion path slows down the aging of NO_2_ inside the coating, thus reducing the sample stratification. As shown in Figure 7b, when the filling amount of f-GO increased to 0.5 wt.%, the impedance modulus of the sample decreased to about 5.99 × 10^6^ Ω·cm^2^. At the same time, $f_{b}$ increases significantly to about 71.1 Hz. It shows that the increase in filler content increases the diffusion channel of NO_2_ gas in the coating to a certain extent. Compared with RTV, although the impedance modulus value is still high, the breakpoint frequency is also high, and the sample stratification is more serious.

**Figure 8:**
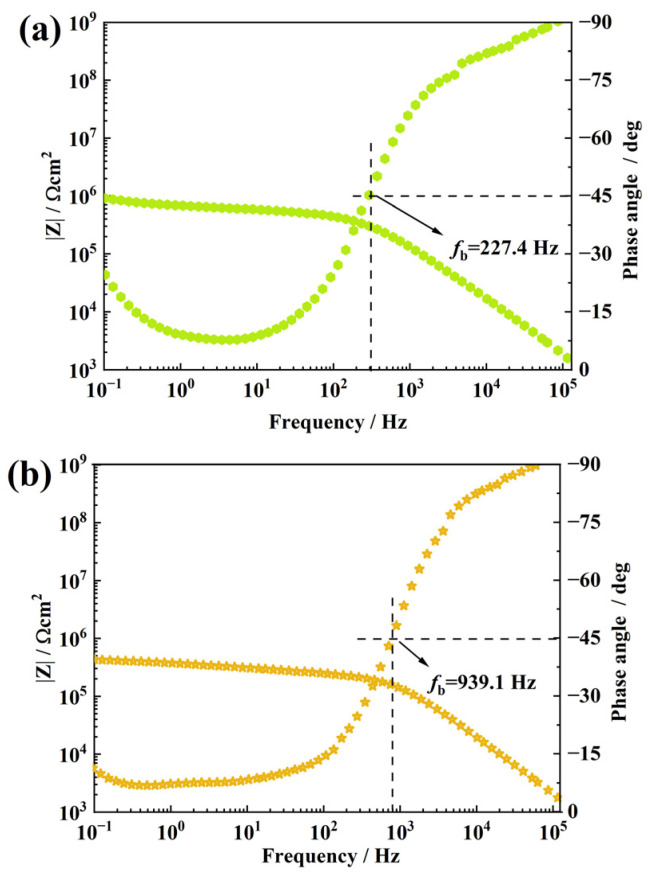
Bode plots of RTV nanocomposite coatings, respectively, filled with 0.7 wt.% (**a**) and 1 wt.% (**b**) f-GO nanosheets after NO2 oxidation.

Figure 8a shows the impedance spectrum of the coating when the content of f-GO increases to 0.7 wt.% after 24 h of NO_2_ aging. Compared with the f-GO nanosheet filled with 0.5 wt.%, the impedance modulus of the sample showed a continuous decrease, which was about 1.04 × 10^6^ Ω·cm^2^. At the same time, $f_{b}$ also increased significantly, about 227.4 Hz, indicating that the sample stratification was more serious. When the filler content increases to 1 wt.%, the impedance modulus decreases more seriously, which decreases to 4.3 × 10^5^ Ω·cm^2^, and $f_{b}$ increases to 939.1 Hz. It shows that an increase in excess filler content greatly reduces the NO_2_ barrier performance of the coating. It is speculated that excessive nano-fillers appear to agglomerate in the coating interior, thus forming a large number of NO_2_ diffusion channels, which makes it easier for NO_2_ to diffuse in the coating interior, thus causing more serious damage to the coating.

**Figure 9:**
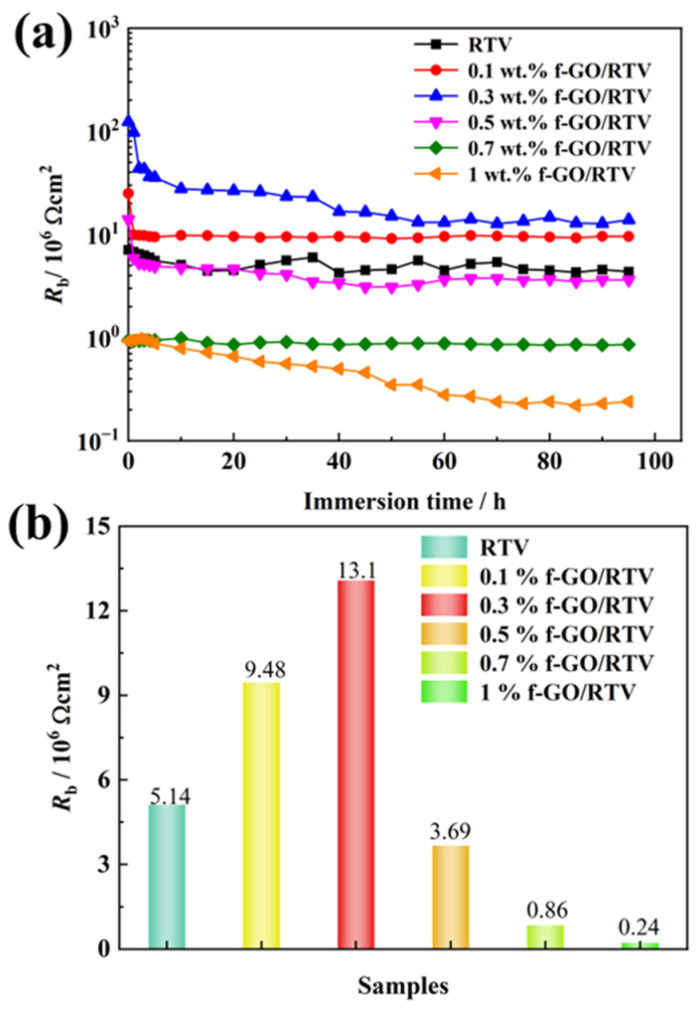
The variation in coating resistance Rb of RTV nanocomposite coatings filled with different contents of f-GO nanosheets after NO2 oxidation: (**a**) with different immersion time, (**b**) the final Rb after immersing for 96 h.

Figure 9 shows the variation in coating resistance (R_b_) with different f-GO nanosheet contents after fitting as a function of soaking time. In order to improve the goodness of fit, the constant phase element is used to replace the capacitor for fitting and then converted into the actual capacitor of the coating through the theoretical numerical formula. As shown in the figure, R_b_ of all coatings showed a rapid decrease in the early stage of immersion and then remained basically unchanged. As can be seen from the figure, R_b_ of pure RTV coating decreases significantly, which is about 5.14 × 10^6^ Ω·cm^2^ after 96 h immersion, which is four orders of magnitude lower than that of the unaged coating. After filling with 0.1 wt.% f-GO nanosheets, the coating resistance increased significantly to about 9.48 × 10^6^ Ω·cm^2^. The results show that the filling of f-GO nanosheets slows down the diffusion rate of NO_2_ gas in the coating. When the content of the nanosheet increases to 0.3 wt.%, R_b_ increases continuously. Compared with pure RTV, R_b_ is nearly two orders of magnitude higher, increasing to about 13.1 × 10^6^ Ω·cm^2^. However, as the content of nanosheets continued to increase, R_b_ decreased significantly after stabilization, especially when the content of nanosheets increased to 1 wt.%; R_b_ was about 2.4 × 10^5^ Ω·cm^2^. The resistance of the coating is one order of magnitude lower than that of pure RTV, which indicates that the excess-filled nanosheet increases the diffusion-free volume of NO_2_ to a certain extent, thus making the resistance of the aging sample decline more seriously. As shown in Figure 9b, with an increase in filler content, R_b_ showed a trend of increasing first and then decreasing; that is, there was an optimal filling ratio of 0.3 wt.% for the f-GO nanosheet in RTV. The increase in R_b_ may be due to the better dispersion of the functionalized nanosheets in the coating, which increases the diffusion path of gas and delays the aging of the coating by NO_2_. However, as the content of the nanosheet continues to increase, the free volume in the coating increases because the nanosheet easily agglomerates, resulting in an increase in the diffusion channels of NO_2_ and a decrease in the tolerance to NO_2_.

**Figure 10:**
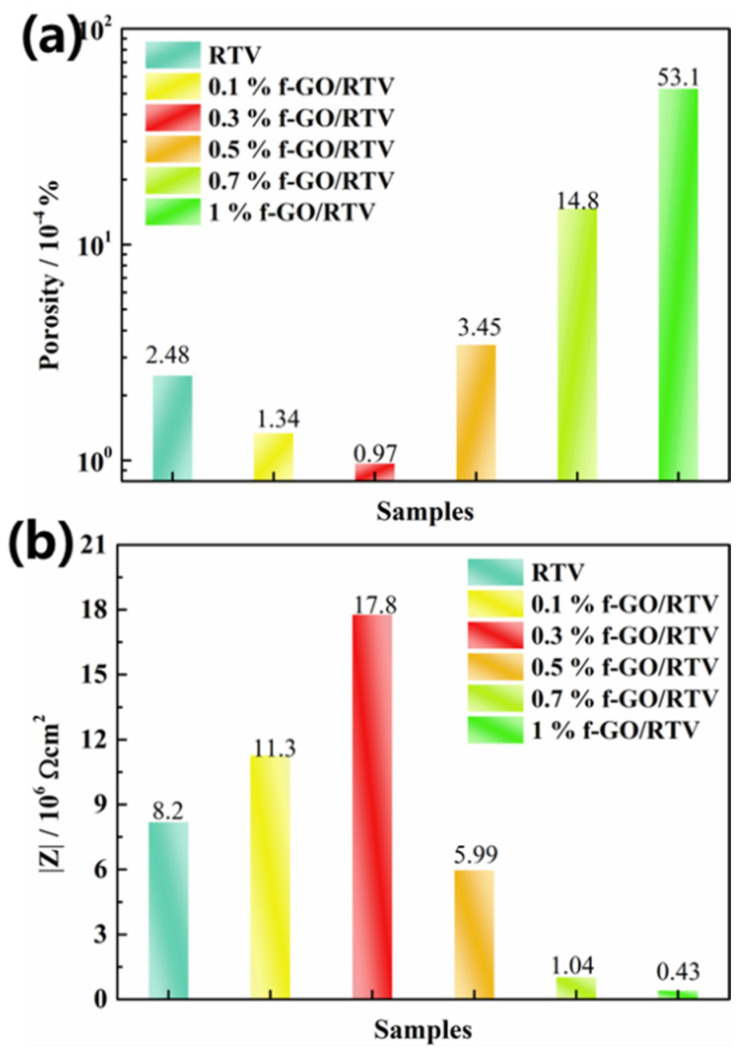
Variations in porosity (**a**) and impedance modules (|Z| at 0.1 Hz) (**b**) of RTV nanocomposite coatings filled with different filler content of f-GO nanosheets after NO_2_ oxidation.

The sample will have defects and water absorption after aging. The change in sample microstructure can be well reflected by calculating the porosity of the sample after soaking for a period of time. The porosity of the sample (P) is defined as the ratio of theoretical resistance at infinite porosity (R_bt_) to the sample resistance obtained from circuit fitting [38]. Figure 10a shows the porosity of RTV coatings filled with different amounts of f-GO nanosheets after NO_2_ aging (citation). The porosity of the coating decreases with an increase in filler content until the content of the nanosheet increases to 0.3 wt.%, and the porosity reaches a minimum value, which is about 0.97 × 10^−4^% and is 40% of the porosity of the pure RTV coating. It is speculated that at this content, silane-modified nanosheets have better dispersion in the coating, which can greatly increase the diffusion path of NO_2_ and delay the damage of NO_2_ to the interior of the coating. However, when the content of f-GO nanosheets continued to increase to 1 wt.%, the porosity of the coating after NO_2_ aging increased significantly to 5.31 × 10^−3^%, which was two orders of magnitude higher than that of the coating filled with 0.3 wt.% f-GO/RTV. It is speculated that at this content stage, excessive nanosheets appear to agglomerate inside the coating, thus increasing the free volume in the coating and providing more channels for NO_2_ diffusion.

In order to show the effect of filler content on the integrity and delamination of nanocomposite coatings, the impedance modulus and breakpoint frequency $f_{b}$ of RTV coatings with different contents of f-GO nanosheets were compared. The impedance modulus value in the Bode diagram (impedance modulus value at 0.1 Hz) is similar to the coating resistance, which can reflect the integrity of the coating. The difference is that the impedance modulus value does not need to be solved, so the impedance modulus value can be very intuitive to judge the water barrier of the coating. As shown in Figure 10b, with an increase in the content of f-GO nanosheets, the impedance modulus of the coating increased first and then decreased. When the content of the nanosheet increases to 0.3 wt.% especially, the impedance modulus reaches 1.78 × 10^7^ Ω cm^2^, which is 2.2-times higher than that of pure RTV (8.2 × 10^6^ Ω cm^2^). These results indicate that appropriate filling of f-GO nanosheets can improve the NO_2_ tolerance of the coating to a certain extent. However, as the filler content continued to increase, the impedance modulus of the coating decreased significantly, especially when the filler content increased to 1 wt.%, where the impedance modulus decreased to about 4.3 × 10^5^ Ω cm^2^, which was two orders of magnitude lower than that of the RTV coating filled with 0.3 wt. % f-GO. The results show that the content of filler is also a key factor affecting the performance of the coating. Due to the small size of nano-fillers, agglomeration can easily occur when the content is high, thus forming more gas diffusion channels inside the coating, which reduces the NO_2_ tolerance of the coating. When the content of f-GO nanosheets reaches 1 wt.%, the coating performance is severely decreased, indicating that the filler content is an important parameter affecting the coating performance, which is consistent with the above resistance analysis of coating samples.

**Figure 11:**
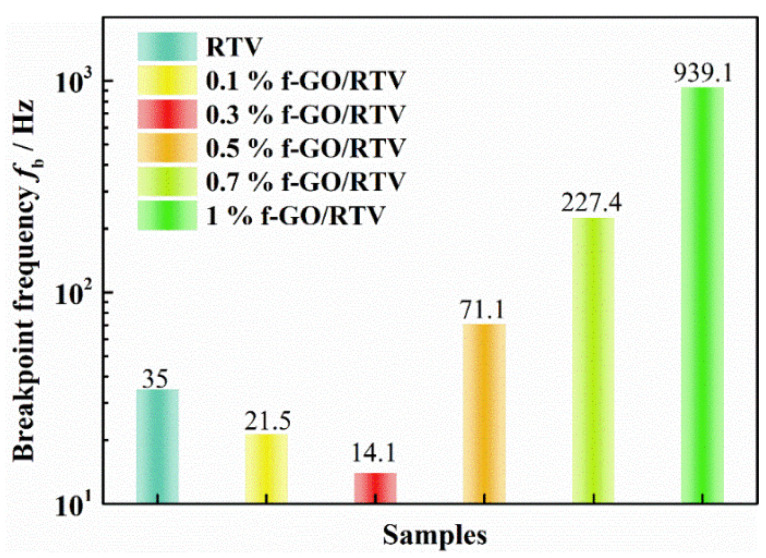
Variations in breakpoint frequency *f_b_* of RTV nanocomposite coatings with different filler content of f-GO nanosheets after NO_2_ oxidation.

As shown in Figure 11, with an increase in f-GO nanosheet content, the $f_{b}$ of the coating decreased first and then increased. Particularly, when the content of f-GO increased to 0.3 wt.%, the breakpoint frequency of the coating decreased to 14.1 Hz, which was much lower than 35 Hz of pure RTV. The results show that filling an appropriate amount of f-GO nanosheet (0.3 wt.%) can block and delay the diffusion of NO_2_ into the coating to a certain extent, so that the layer between the coating and the substrate is smaller. However, the breakpoint frequency of the coating increases obviously with an increase in the content of the nanosheet. When the content increased to 1 wt.%, the $f_{b}$ of the coating increased to 939.1 Hz, which was 66.6-times that of the coating filled with 0.3 wt.% nanosheets. The results indicate that more NO_2_ diffusion channels are formed by the filling of excessive functional groups, and the water barrier performance of the coating decreases more significantly after NO_2_ aging. At the same time, 0.3 wt.% GO/RTV shows the lowest delamination and the best integrity after oxidation by NO_2_, with excellent damage protection performance. Therefore, the content of the nanosheet is an important parameter affecting the performance of the coating.

**Figure 12:**
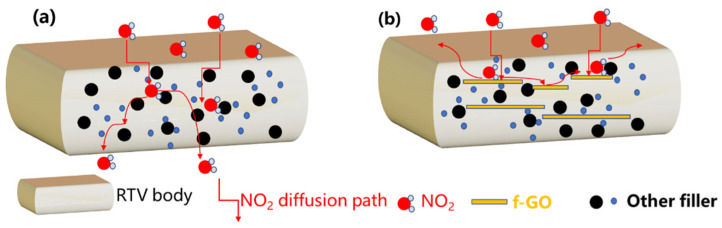
The physical barrier mechanism diagram of RTV (**a**) and f-GO/RTV (**b**).

Based on the above analysis of water resistance results, we gave a possible f-GO blocking NO_2_ model. The physical barrier mechanism diagram can be seen in Figure 12. Firstly, silane grafting increased the interface compatibility of two-dimensional nanosheets in RTV. At the same time, the functionalized nanosheets acted as a cross-linking and strengthening silicone rubber organic matrix, reducing the free volume of NO_2_ diffusion. Secondly, the silican-modified nanosheets enhance the NO_2_ barrier property of silicone rubber from two aspects: the successful grafting of a silane enhances the interface compatibility between the nanosheets and silicone rubber and reduces the free volume of NO_2_ gas diffusion; the high aspect ratio of its graphene-like structure is utilized to provide physical barrier properties. Because the added f-GO filler blocks the diffusion of NO_2_ in RTV, under the same aging condition, f-GO/RTV is less damaged, which shows that it has a higher impedance modulus and lower breakpoint frequency in the EIS test, and it still has good water barrier performance.

## 4. Conclusions

In summary, this paper successfully prepared f-GO that is modified by TEOS and APTES, which can be dispersed well in an RTV coating. After the aging of NO_2_ for 24 h, the impedance modulus |Z| and resistance R_b_ of the RTV coating first increase and then decrease with an increase in f-GO addition. On the contrary, the porosity P and the broken frequency $f_{b}$ of the RTV coating first decrease and then increase with an increase in f-GO addition. All of them have the best value at a 0.3 wt.% f-GO filler content, which indicates that the f-GO/RTV coating has the best water resistance performance. The results show that the silane-modified nanosheets can enhance the NO_2_ barrier of silicone rubber, mainly from two aspects. Firstly, the successful grafting of a silane enhances the interface compatibility between the GO nanosheets and silicone rubber and reduces the free volume of NO_2_ gas diffusion. The second is to provide physical barrier properties by taking advantage of the high aspect ratio of its lamella structure. Herein, the strategy developed in this paper offers a new point for polymer systems to enhance chemical oxidative damage protection properties using 2D heteroatom-containing nanosheets materials, such as f-GO. This proposed method not only improves the safety of silicone rubber insulator operation but also greatly reduces the cost of power grid units.

## Figures and Tables

**Table 1:** The elemental composition of GO and f-GO nanosheets.

Samples	C/at%	N/at%	O/at%	Si/at%
GO	66.56	/	33.44	/
f-GO	32.38	1.9	39.57	26.15

## Data Availability

Raw data are available upon request.
